# Burden of illness in Rett syndrome: initial evaluation of a disorder-specific caregiver survey

**DOI:** 10.1186/s13023-024-03313-8

**Published:** 2024-08-13

**Authors:** Walter E. Kaufmann, Alan K. Percy, Jeffrey L. Neul, Jenny Downs, Helen Leonard, Paige Nues, Girish D. Sharma, Theresa E. Bartolotta, Gillian S. Townend, Leopold M. G. Curfs, Orietta Mariotti, Claude Buda, Heather M. O’Leary, Lindsay M. Oberman, Vanessa Vogel-Farley, Katherine V. Barnes, Christopher U. Missling

**Affiliations:** 1 Anavex Life Sciences Corp, New York, NY USA; 2 Emory University School of Medicine, Atlanta, GA USA; 3 University of Alabama at Birmingham Heersink School of Medicine, Birmingham, AL USA; 4 Vanderbilt University Medical Center, Nashville, TN USA; 5 Telethon Kids Institute, The University of Western Australia, Nedlands, WA Australia; 6 International Rett Syndrome Foundation (IRSF), Cincinnati, OH USA; 7 Rush University Medical Center, Chicago, IL USA; 8 Monmouth University, West Long Branch, NJ USA; 9 Maastricht University, Maastricht, The Netherlands; 10 Pro Rett Ricerca, Sermide E Felonica, Mantua, Italy; 11 Rett Syndrome Association of Australia (RSAA), Grovedale, VIC Australia; 12 National Institute of Mental Health Intramural Research Program, National Institutes of Health, Bethesda, MD USA

**Keywords:** Rett syndrome, Quality of life, Intellectual disability, Caregiver, Parent-proxy report

## Abstract

**Background:**

Rett syndrome (RTT) is a severe X-linked neurodevelopmental disorder associated with multiple neurologic impairments. Previous studies have shown challenges to the quality of life of individuals with RTT and their caregivers. However, instruments applied to quantify disease burden have not adequately captured the impact of these impairments on affected individuals and their families. Consequently, an international collaboration of stakeholders aimed at evaluating Burden of Illness (BOI) in RTT was organized.

**Methods:**

Based on literature reviews and qualitative interviews with parents of children and adults with RTT, a caregiver questionnaire was constructed to evaluate 22 problems (inclusive of core characteristics, functional impairments, and comorbidities) often experienced with RTT, rated mainly with a 5-level Likert scale. The questionnaire was administered anonymously online to an international sample of 756 caregivers (predominantly parents) of girls and women with RTT. Descriptive statistics were used to identify problems of high frequency and impact on affected individuals and caregivers. Chi-square tests characterized the relationship between problem severity and impact responses, while nonparametric ANOVAs of raw and z-score adjusted scores identified agreement between severity and impact on individual and caregiver. Secondary inferential tests were used to determine the roles of age, clinical type, and country of residence on BOI in RTT.

**Results:**

There was variability in reported frequency of problems, with the most prevalent, severe and impactful being those related to the core features of RTT (i.e., communication and fine and gross motor impairments). Chi-square analyses demonstrated interdependence between severity and impact responses, while ANOVAs showed that many problems had disproportionately greater impact than severity, either on affected individuals (e.g., hand stereotypies) or their caregivers (e.g., sleep difficulties, seizures, pain, and behavioral abnormalities). With certain exceptions (e.g., breath-holding, seizures), age, clinical type, or country of residence did not influence these BOI profiles.

**Conclusions:**

Our data demonstrate that core features and related impairments are particularly impactful in RTT. However, problems with mild severity can also have disproportionate impact on affected individuals and, particularly, on their caregivers. Future analyses will examine the role of factors such as treatment outcomes, healthcare services, and healthcare provider’s perspectives, in these BOI profiles.

**Supplementary Information:**

The online version contains supplementary material available at 10.1186/s13023-024-03313-8.

## Background

Rett Syndrome (RTT; OMIM 312750) is a rare X-linked neurodevelopmental disorder that occurs predominantly in girls and women with an incidence of approximately 1 in 10,000 female births worldwide [1, 2]. Most individuals with RTT (> 96%) carry a pathogenic variant in the methyl-CpG-binding protein 2 (*MECP2*) gene, which encodes the transcriptional regulator MeCP2 [3, 4]. The disorder is characterized by progression of neurologic impairment throughout development into adulthood [5, 6]. Most individuals with RTT appear to develop normally until around 6–18 months of age, after which they experience a period of regression characterized by loss of spoken language and fine motor skills. This regression, in conjunction with impairment in ambulation and development of hand stereotypic movements, constitute the core diagnostic features of the disorder [5]. Recovery of language and fine motor skills is limited as is the further development of gross motor skills [5, 6]. In addition to these impairments, other neurologic and systemic manifestations frequently develop [7]; these include seizures [8], sleep problems [9], breathing abnormalities [10], aberrant behaviors [11], musculoskeletal abnormalities (e.g., scoliosis) [12, 13], and gastrointestinal dysfunction [14]. Although the clinical manifestations of RTT reach relative stability after childhood, further decline in multiple functions may become evident at older ages. Adulthood is a period characterized by limited motor (e.g., emergence of Parkinsonian features) and communication abilities, as well as for the development of internalizing behavioral abnormalities (e.g., depression-like symptoms) [11, 15–17].

Thanks to advances in medical and allied health care, including better recognition of factors affecting morbidity and mortality, many individuals with RTT survive into their 50 s [18–20]. However, ongoing functional deficits and comorbidities experienced may pose significant physical, psychological, social and financial burden on affected individuals and their caregivers. Several studies examining the impact of RTT identified challenges to quality of life of affected individuals [21–23], their siblings [24, 25], and their caregivers [25–31]. These investigations have identified multiple factors affecting outcomes and quality of life in RTT. For affected individuals, these include ability to communicate and ambulate, feeding skills, age of onset of hand stereotypies, severity of seizures, sleep problems and behavioral abnormalities [21–23, 32–36]. The impact on the caregiver’s physical and mental well-being is dependent on, among others, the severity of the child’s physical and behavioral impairments, in particular feeding difficulties; caregiver age and demands; financial challenges; and challenges to family functioning [25–28, 31]. Findings on maternal mental health suggest an increased risk of anxiety but they are not conclusive about depression [25–27]. Of interest is the observation that caregiver mental health is more affected than physical quality of life, and that this profile does not change over a 5-year period [28]. A small study on siblings of girls and women with RTT showed relatively good psychological adjustment, in comparison with population norms [24], while another larger investigation, contrasting the impact with that on siblings of children with Down syndrome, found both benefits and disadvantages for the RTT group [24, 25].

Despite this increasing literature, many questions remain about the burden of RTT on affected individuals and their families. Previous studies have applied standardized instruments (e.g., Child Health Questionnaire 50) which are not validated for evaluating a population like RTT, with severe communication, motor impairments and other unique clinical features (e.g., “Rett episodes”). Moreover, many surveys have been implemented with relatively small caregiver samples that may not have captured the population-level variability of the disorder and familial experience. Recently, domains of quality of life important for children [37] and adults with RTT have been explored [38]. Accordingly, new instruments and analytical strategies are being developed to investigate the impact of RTT on individuals [37, 39] and their caregivers [40].

One of these initiatives, reported in the present study, was to implement a comprehensive, large-scale, international study to investigate RTT specific issues. To accomplish these goals, RTT stakeholders representing affected families, clinicians, researchers, and drug developers, in the USA, Europe, and Australia, joined efforts in a “Burden of Illness” project. Here, we report initial results from the caregiver survey on BOI in females with RTT to identify (1) problems (core features, functional impairments, comorbidities) of greater frequency and impact, (2) relationships between severity of a problem, as assessed by caregivers, and its impact on individuals and caregivers, (3) agreement between impact on affected individual and impact on caregiver, and (4) roles of age, clinical type, and country of residence on BOI in RTT.

## Methods

### Data sources

Caregivers of female and male individuals with RTT, both children and adults, from the USA, United Kingdom, Italy, Germany, and Australia were invited to participate by their countries’ advocacy groups. In Australia, most participants were recruited from the Australian Rett Syndrome Database [41]. For this study, caregivers were defined as those who reported being 18 years or older and spending at least 10 h per week caring for an individual with RTT. No clinical type (diagnosis of classic or atypical RTT) was required for participation. Because of their different prevalence and clinical features, here we report only on girls and women with caregiver-reported RTT. A separate analysis will investigate the survey data of caregivers of male individuals. As shown in Table 1 most caregivers (96%) were parents of affected individuals. A small proportion (4%) of surveys were completed by grandparents, siblings, and paid caregivers. A total of 756 caregivers provided verified surveys of female individuals with RTT. Survey verification was performed by reviewing responses and open text entries to validate that the data provided was complete and legitimate. Surveys that were identified as being completed in an erroneous manner (i.e., user acceptance testing responses, duplicate entries, nonsensical open text fields with clearly invalid responses) were removed from the analyses (N = 326). Profiles of affected individuals and their caregivers participating in the present study are shown in Table 1.

**Table 1:** Demographic and clinical features of affected individuals and caregivers

	Age (years)		Diagnosis age (years)		Full dataset		Diagnosis					
							Classic Rett syndrome		Atypical Rett syndrome		Don’t know	
	Mean	(SD)	Mean	(SD)	N	(%)	N	(%)	N	(%)	N	(%)
Total	16.72	10.67	4.30	4.65	756	100	519	69	143	19	94	12
Age	Mean	(SD)	Mean	(SD)	N	(%)	N	(%)**	N	(%)**	N	(%)**
Child (< 12 years)	6.82	2.87	2.75	1.36	303	40.1	214	70.6	48	15.8	41	13.5
Adolescent (12–18 years)	15.10	1.72	3.53	2.77	152	20.1	104	68.4	33	21.7	15	9.9
Adult (> 19 years)	27.50	7.71	6.24	6.50	301	39.8	201	66.8	62	20.6	38	12.6
Mutation												
*MECP2* Mutation	15.83	10.34	4.11	4.53	673	89.0	486	72.2	113	16.8	74	11.0
No *MECP2* Mutation Identified	23.62	10.01	6.03	5.01	31	4.1	6	19.4	21	67.7	4	12.9
Don’t know	24.10	11.03	5.75	5.59	52	6.9	27	51.9	9	17.3	16	30.8
Region												
United States	16.70	11.13	4.01	4.14	415	54.9	282	68.0	86	20.7	47	11.3
Europe*	16.24	10.51	4.58	5.25	259	34.3	193	74.5	42	16.2	24	9.3
Australia	18.36	8.49	4.86	5.07	82	10.8	44	53.7	15	18.3	23	28.0
Caregiver												
Parent	16.75	10.57	4.31	4.57	728	96.3	502	69.0	140	19.2	86	11.8
Grandparent	9.81	9.93	2.32	0.98	14	1.9	7	50.0	3	21.4	4	28.6
Sibling	36.08	15.27	15.72	16.60	3	0.4	1	33.3	0	0.0	2	66.7
Paid caregiver	17.95	10.59	3.24	3.85	11	1.5	9	81.8	0	0.0	2	18.2

*Europe includes United Kingdom (N = 103), Germany (N = 100), and Italy (N = 56)

**Percent of respective analysis group when caregiver selected the diagnosis of “Classic”, “Atypical”, or “Don’t know”

Approval to conduct the study was obtained from ethics committees in each country and either written or electronic informed consent was obtained for all participants. Surveys were completed anonymously, no identifying information was collected, and data were maintained confidential in accordance with the ethics protocol.

### Procedure

The caregiver BOI survey was developed following literature review, input from an advisory board of experts (both clinicians and caregivers), and concept elicitation (qualitative) interviews with 15 parents of children and adults with RTT. Survey development included caregiver cognitive debriefing for evaluating the relevance and comprehension of the sections and questions. The questionnaire included 138 questions covering 22 characteristic problems (core clinical features, functional impairments, comorbidities), spanning 15 domains (Table 2). Additional sections on quality of life, healthcare resource utilization, and general impact on caregivers (health and relationships, work productivity, financial impact), comprising an additional 38 questions were not analyzed in the present study because of its focus on the impact of specific problems in RTT. Responses to these questions will be included in subsequent analyses and published in a separate paper. Ratings of problem severity were based on caregivers’ experiences during the previous 4 weeks. Problem impact was assessed by the caregiver; including both impact on the affected individual’s ability to participate in daily activities (Impact on individual) and impact on the caregiver (e.g., physical or emotional well-being). Example sections of the caregiver survey are presented in Supplementary Material.

**Table 2:** Profile of responses to severity, impact on individual, and impact on caregiver questions

Domain	Problem (order of survey presentation)	% Reporting the problem	Question	% of Responses at 2 highest levels in those reporting problem	Raw scores		Z-scores	
					Median	Mean (SD)	Median	Mean (SD)
Breathing	Breath-Holding	56.6	Severity	28.7	3	2.74 (1.2)	− 0.36	− 0.51 (0.69)
			Impact-individual	18.0	2	2.34 (1.21)	− 1.15	− 0.9 (0.92)
			Impact-caregiver	27.3	3	2.67 (1.22)	− 0.39	− 0.64 (0.93)
	Hyperventilation^e^	35.4	Severity	26.5	3	2.76 (1.08)	− 0.36	− 0.5 (0.62)
			Impact-individual	23.9	2	2.54 (1.18)	− 1.15	− 0.74 (0.9)
			Impact-caregiver	27.2	3	2.71 (1.15)	− 0.39	− 0.61 (0.87)
	Air swallowing	41.5	Severity	34.1	3	2.99 (1.17)	− 0.36	− 0.36 (0.67)
			Impact-individual	20.7	2	2.52 (1.19)	− 1.15	− 0.76 (0.9)
			Impact-caregiver	28.7	3	2.71 (1.27)	− 0.39	− 0.61 (0.96)
Hand use	**Functional hand use**^**c, f**^	95.9	Severity	76.0	5	5.1 (0.92)	0.79	0.84 (0.53)
			Impact-individual	90.1	5	4.59 (0.78)	1.13	0.82 (0.59)
			Impact-caregiver	72.7	4	4.03 (1.12)	0.37	0.39 (0.85)
Involuntary movements	**Hand stereotypies**	97.8	Severity	69.7	4	3.84 (1.1)	0.21	0.12 (0.63)
			Impact-individual	74.3	5	4.11 (1.12)	1.13	0.45 (0.85)
			Impact-caregiver	57.4	4	3.62 (1.26)	0.37	0.08 (0.96)
Gastrointestinal	Constipation^d^	79.0	Severity	41.0	3	3.26 (1.08)	− 0.36	− 0.21 (0.62)
			Impact-individual	26.8	3	2.79 (1.21)	− 0.39	− 0.55 (0.92)
			Impact-caregiver	44.6	3	3.29 (1.23)	− 0.39	− 0.17 (0.94)
	Gastroesophageal reflux	37.6	Severity	27.1	3	2.84 (1.08)	− 0.36	− 0.45 (0.62)
			Impact-individual	23.6	2	2.67 (1.16)	− 1.15	− 0.65 (0.88)
			Impact-caregiver	34.2	3	2.99 (1.22)	− 0.39	− 0.4 (0.93)
Feeding	Oral feeding^f^	68.4	Severity	29.6	4	4.14 (1.01)	0.21	0.29 (0.58)
			Impact-individual	35.6	3	2.92 (1.25)	− 0.39	− 0.45 (0.95)
			Impact-caregiver	54.4	4	3.51 (1.23)	0.37	0 (0.94)
Scoliosis	Scoliosis^d^	59.3	Severity	24.6	3	2.67 (1.22)	− 0.36	− 0.55 (0.7)
			Impact-individual	27.9	3	2.67 (1.32)	− 0.39	− 0.64 (1)
			Impact-caregiver	37.5	3	2.96 (1.33)	− 0.39	− 0.42 (1.02)
Communication	Understanding^a, e, f^	48.8	Severity	48.5	4	4.14 (0.99)	0.21	0.29 (0.56)
			Impact-individual	79.1	5	4.22 (0.97)	1.13	0.53 (0.74)
			Impact-caregiver	74.8	4	4.12 (1.08)	0.37	0.46 (0.82)
	Nonverbal self-expression^e, f^	62.8	Severity	25.7	4	3.97 (0.85)	0.21	0.2 (0.49)
			Impact-individual	81.7	5	4.32 (0.93)	1.13	0.61 (0.71)
			Impact-caregiver	76.4	5	4.18 (1.09)	1.13	0.5 (0.83)
	**Verbal self-expression**^**f**^	92.7	Severity	80.7	6	5.28 (0.9)	1.36	0.95 (0.51)
			Impact-individual	88.2	5	4.5 (0.88)	1.13	0.75 (0.67)
			Impact-caregiver	80.9	5	4.32 (1.05)	1.13	0.61 (0.8)
Mobility	**Standing unsupported**^**e, f**^	75.7	Severity	77.6	6	5.34 (1.04)	1.36	0.98 (0.59)
			Impact-individual	90.0	5	4.59 (0.77)	1.13	0.82 (0.59)
			Impact-caregiver	87.1	5	4.49 (0.91)	1.13	0.74 (0.69)
	**Walking with assistance**^**b, f**^	69.7	Severity	60.2	5	4.86 (1.15)	0.79	0.71 (0.66)
			Impact-individual	91.8	5	4.64 (0.73)	1.13	0.86 (0.55)
			Impact-caregiver	88.2	5	4.54 (0.86)	1.13	0.78 (0.65)
	**Walking independently**^**e, f**^	82.4	Severity	78.0	6	5.37 (1.01)	1.36	1 (0.58)
			Impact-individual	88.3	5	4.53 (0.82)	1.13	0.77 (0.62)
			Impact-caregiver	86.0	5	4.46 (0.93)	1.13	0.72 (0.71)
Sleep	Sleep difficulties^c^	71.8	Severity	37.6	3	3.13 (1.03)	− 0.36	− 0.28 (0.59)
			Impact-individual	37.0	3	3.18 (1.12)	− 0.39	− 0.25 (0.85)
			Impact-caregiver	63.0	4	3.85 (1.12)	0.37	0.25 (0.85)
Epilepsy	Seizures	36.8	Severity	30.2	2	2.67 (1.44)	− 0.93	− 0.55 (0.82)
			Impact-individual	49.3	3	3.45 (1.27)	− 0.39	− 0.05 (0.96)
			Impact-caregiver	65.1	4	3.85 (1.16)	0.37	0.26 (0.89)
Rett episodes	Rett episodes	79.2	Severity	25.2	3	2.68 (1.14)	− 0.36	− 0.54 (0.65)
			Impact-individual	29.0	3	2.81 (1.22)	− 0.39	− 0.54 (0.93)
			Impact-caregiver	37.6	3	3.08 (1.26)	− 0.39	− 0.33 (0.96)
Dystonia	Dystonia^c, d^	53.0	Severity	22.2	3	2.79 (1.07)	− 0.36	− 0.48 (0.61)
			Impact-individual	32.9	3	3.02 (1.15)	− 0.39	− 0.38 (0.88)
			Impact-caregiver	39.4	3	3.20 (1.2)	− 0.39	− 0.24 (0.91)
Pain	Pain^c^	73.0	Severity	20.1	2.5	2.57 (1.1)	− 0.65	− 0.61 (0.63)
			Impact-individual	31.3	3	2.93 (1.2)	− 0.39	− 0.45 (0.92)
			Impact-caregiver	57.6	4	3.62 (1.22)	0.37	0.08 (0.93)
Behaviors	Behavioral abnormalities^c^	79.9	Severity	34.3	3	3.06 (1.07)	− 0.36	− 0.33 (0.61)
			Impact-individual	39.9	3	3.23 (1.1)	− 0.39	− 0.22 (0.84)
			Impact-caregiver	63.9	4	3.86 (1.12)	0.37	0.26 (0.85)
Self-care	Self-care^e, f^	98.5	Severity	98.0	6	5.91 (0.38)	1.36	1.31 (0.22)
			Impact-individual	80.7	5	4.3 (1.17)	1.13	0.6 (0.89)
			Impact-caregiver	87.1	5	4.5 (0.97)	1.13	0.74 (0.74)

Core features and related impairments in bold

^a^Chi-square severity versus impact individual and severity versus impact caregiver not significant

^b^Friedman’s ANOVA not significant

^c^Dunn Bonferroni’s post hoc severity versus impact-individual not significant

^d^Dunn Bonferroni’s post hoc severity versus impact-caregiver not significant

^e^Dunn Bonferroni’s post hoc impact-individual versus impact-caregiver not significant

Surveys were completed via web interface. Analyses were performed on surveys from caregivers with a valid and unique entry (IP address), who met the definition of caregiver, and reported that the affected individual was female. If there were duplicate entries for a single caregiver, the most complete surveys were included and the least complete were discarded. For the present study, we excluded a few surveys (n = 14) from caregivers who reported that the affected individual had either a *FOXG1* or a *CDKL5* variant, since pathogenic variants of these genes are now considered distinctive disorders [42, 43].

Questions on impact on individual, impact on caregiver and half of those assessing severity, were scored using a 5-level Likert scale, ranging from very mild/low to very severe/high. For severity items evaluating episodic manifestations (i.e., pain, seizures, “Rett episodes”) a ‘None in the past 4 weeks’ option was added below the very mild/low option. Severity items evaluating functional impairments were scored using 6 levels, ranging from excellent to unable. The survey was translated from English into German, Italian, and Spanish following the International Society for Pharmacoeconomics and Outcomes Research (ISPOR) task force guidance and were administered online.

### Data analysis

The primary analyses included data on demographics and sample characteristics and the questions on severity, impact on individual, or impact on caregiver for the entire sample. The full dataset of 756 surveys was divided into groups for secondary analyses based on age, clinical type, and country or region of residence: children (individuals younger than 12 years), adolescents (individuals between 12 and 18 years), and adults (individuals older than 18 years); clinical type (diagnosis of classic or atypical RTT); residential country/region: Australia, Europe (European countries were grouped), and the United States. Because most caregivers reported a “known” *MECP2* pathogenic variant (~ 89%), this parameter was not included in the analyses.

We calculated raw scores from the caregivers’ categorical responses to all questions, assigning 0 to “none”, 1 through 5 to “very mild/low” through “very severe/high”, and 6 to “unable”. To control for heterogeneity between questions with 5 or more severity levels, we standardized responses across the entire set of 22 problems by calculating z-scores. As the analyses required a comparison of severity and impact on the individual and caregiver, for each problem we only analyzed surveys where the caregiver confirmed that the individual was affected by the problem and responded to the severity, impact on individual, and impact on caregiver questions. We excluded surveys where the caregiver confirmed that the individual was affected by the problem but one or more of these questions were not responded to (n = 0–11, depending on the problem).

**Fig. 1:**
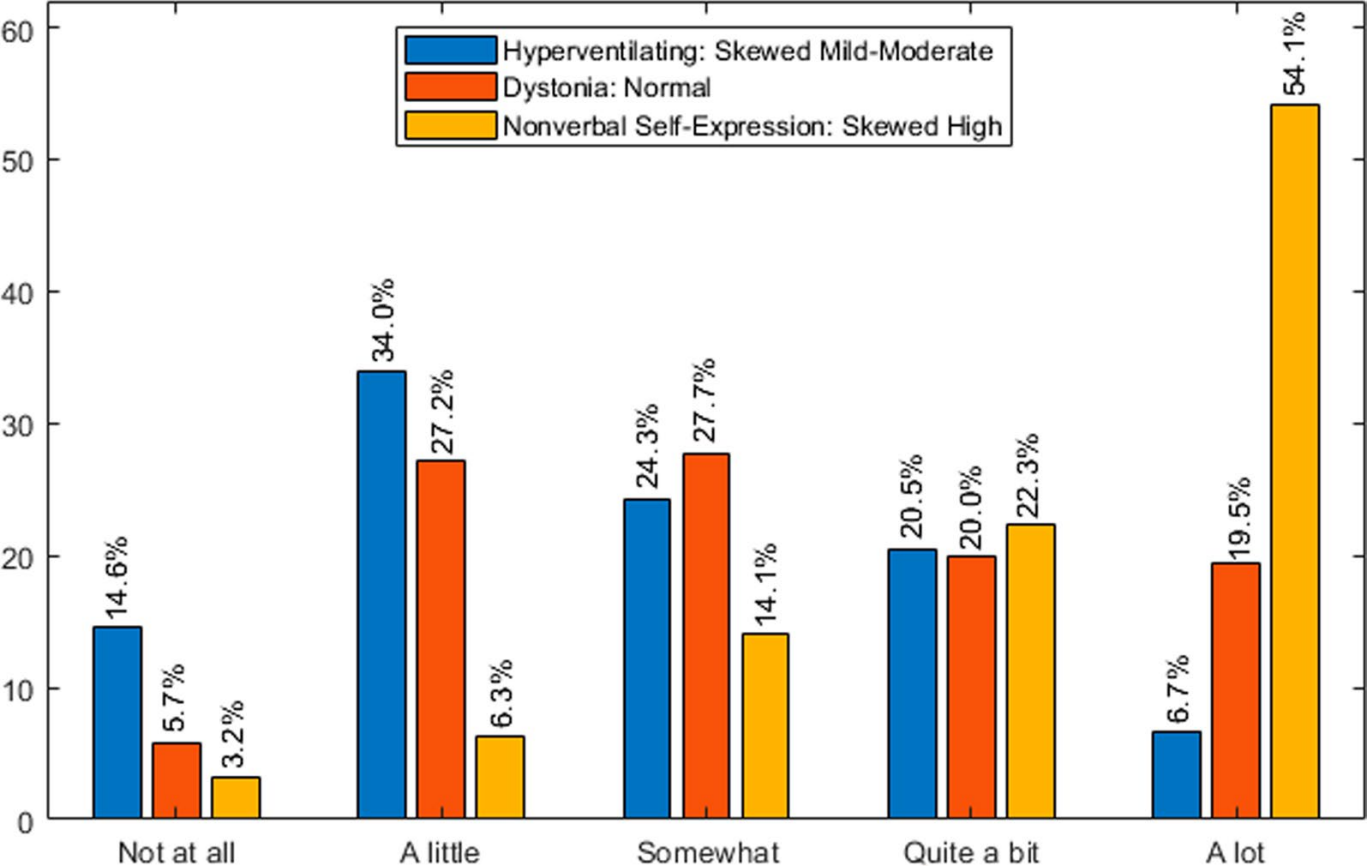
Patterns of distribution of responses in caregiver survey: three examples of impact caregiver frequency histograms

Descriptive analyses depicted in Table 2 include overall frequency of problems (percentage of caregivers reporting the problem), distribution of categorical responses (i.e., percentage of two highest-level responses in those reporting the problem), and median, mean, and standard deviation of raw scores and z-scores for each severity and impact question. In addition, we profiled descriptive data as frequency histograms (Fig. 1). Since severity and impact questions for each of the 22 problems were answered by the same caregiver, we examined interdependence of responses by the chi-square test. Considering that most of chi-square tests were significant, indicating that most responses for a given problem were statistically dependent on each other, we further investigated the nature of their relationship by comparing z-scores on severity, impact on individual, and impact on caregiver for each problem. Given the lack of normal distribution and the relatedness of scores, we conducted ANOVAs using the nonparametric Friedman’s test. This was followed by Dunn-Bonferroni post hoc tests correcting for within-problem multiple comparisons. Since each problem was deemed to be an independent subject of investigation, no multiple comparison corrections across the dataset (i.e., between problems) were conducted. ANOVAs were performed on the full dataset (primary analyses) and on the groups mentioned above (secondary analyses) and illustrated in Tables 2 and 3 for the entire cohort and Tables 4 and 5 for the group analyses. ANOVA summary tables (Tables 3, 4, 5) depict significant mean differences between severity and/or impact scores for each problem. For these summaries, non-significant post hoc differences were considered as approximately equal means. Analyses and histograms were performed using Matlab 9.7.0 (R2019b; The Mathworks Inc., Natick, Massachusetts) and the IBM SPSS Statistics version 29.0.1.0 (171) software (https://www.ibm.com/products/spss-statistics).

**Table 3:** Primary analyses full dataset: relationships between severity, impact on individual, and impact on caregiver

	Severity > impact	Severity = impact	Impact > severity	Total
Impact individual > impact caregiver	**Verbal self-expression^**	**Functional hand use^**	**Hand stereotypies**	3
	1	1	1	
Impact individual = impact caregiver	Hyperventilation			6
	**Standing unsupported^**			
	**Walking independently^**	**Walking with assistance^**	Nonverbal self-expression^	
	Self-care^			
	4	1	1	
Impact caregiver > impact individual			Gastroesophageal reflux	12
			Sleep difficulties	
	Breath-holding	Constipation	Seizures	
	air swallowing	Scoliosis	Rett episodes	
	oral feeding^		Dystonia	
			Pain	
			Behavioral abnormalities	
	3	2	7	
Total	8	4	9	21

Core features and related impairments in bold

Relationship between the severity and impact could not be determined for “Understanding”

^Impairment in function

**Table 4:** Secondary analyses age: relationships between severity, impact on individual and impact on caregiver

	Severity > impact	Severity = impact	Impact > severity	Total
A. Child (< 12 years)				
Impact individual > impact caregiver		**Functional hand use^**	**Hand stereotypies**	2
	0	1	1	
Impact individual = impact caregiver	Hyperventilation			9
	Air swallowing			
	**Verbal self-expression^**		Understanding^	
	**Standing unsupported^**	**Walking with assistance^**	Nonverbal self-expression^	
	**Walking independently^**			
	Self-care^			
	6	1	2	
Impact caregiver > impact individual			Scoliosis	11
		Breath-holding	Sleep difficulties	
		Constipation	Seizures	
		Gastroesophageal reflux	Rett episodes	
		Oral feeding^	Dystonia	
			Pain	
			Behavioral abnormalities	
	0	4	7	
Total	6	6	10	22
B. Adolescent (12–18 years)				
Impact individual > impact caregiver		**Functional hand use^**	**Hand stereotypies**	2
	0	1	1	
Impact individual = impact caregiver	Breath-holding			14
	Hyperventilation	Gastroesophageal reflux		
	Air swallowing	Understanding^	Nonverbal self-expression^	
	**Verbal self-expression^**	**Walking with assistance^**	Seizures	
	**Standing unsupported^**	Rett episodes		
	**Walking independently^**	Dystonia		
	Self-care^			
	7	5	2	
Impact caregiver > impact individual			Scoliosis	6
		Constipation	Sleep difficulties	
		Oral feeding^	Pain	
			Behavioral abnormalities	
	0	2	4	
Total	7	8	7	22
C. Adult (> 18 years)				
Impact individual > impact caregiver	**Functional hand use^**		**Hand stereotypies**	4
	**Verbal self-expression^**		Nonverbal self-expression^	
	2	0	2	
Impact individual = impact caregiver	Hyperventilation			8
	Air swallowing			
	Scoliosis			
	**Standing unsupported^**	Dystonia	Seizures	
	**Walking independently^**			
	Self-care^			
	6	1	1	
Impact caregiver > impact individual	Breath-holding	Constipation	Sleep difficulties	8
	Oral feeding^	Gastroesophageal reflux	Pain	
		Rett episodes	Behavioral abnormalities	
	2	3	3	
Total	10	4	6	20

Core features and related impairments in bold

Relationship between severity and impact could not be determined for “Understanding” and “Walking with Assistance”

^Impairment in function

**Table 5:** Secondary analyses residential region: relationships between severity, impact on individual, and impact on caregiver

	Severity > impact	Severity = impact	Impact > severity	Total
A. United States of America				
Impact individual > impact caregiver		**Functional hand use^**		1
	0	1	0	
Impact individual = impact caregiver	Hyperventilation			8
	**Verbal self-expression^**	**Walking with assistance^**		
	**Standing unsupported^**	Dystonia	Nonverbal self-expression^	
	**Walking independently^**			
	Self-care^			
	5	2	1	
Impact caregiver > impact individual		**Hand stereotypies**	Sleep difficulties	12
	Breath-holding	Constipation	Seizures	
	Air swallowing	Gastroesophageal reflux	Rett episodes	
		Oral feeding^	Pain	
		Scoliosis	Behavioral abnormalities	
	2	5	5	
Total	7	8	6	21
B. Europe				
Impact individual > impact caregiver		**Functional hand use^**	**Hand stereotypies**	2
	0	1	1	
Impact individual = impact caregiver	Air swallowing			10
	Gastroesophageal reflux			
	**Verbal self-expression^**	Hyperventilation		
	**Standing unsupported^**	**Walking with assistance^**	Nonverbal self-expression^	
	**Walking independently^**	Dystonia		
	Self-care^			
	6	3	1	
Impact caregiver > impact individual		Breath-holding	Sleep difficulties	8
		Constipation	Rett episodes	
		Oral feeding^	Pain	
		Scoliosis	Behavioral abnormalities	
	0	4	4	
Total	6	8	6	20
C. Australia				
Impact individual > impact caregiver		**Functional hand use^**	**Hand stereotypies**	2
	0	1	1	
Impact individual = impact caregiver		Hyperventilation		8
	Breath-holding	Scoliosis		
	Air swallowing	Nonverbal self-expression^		
	Self-care^	Rett episodes		
		Dystonia		
	3	5	0	
Impact caregiver > impact individual			Sleep difficulties	4
		Constipation	Pain	
			Behavioral abnormalities	
	0	1	3	
Total	3	7	4	14

(A) Relationship between the severity and impact could not be determined for “Understanding”. (B) Relationship between the severity and impact could not be determined for “Understanding” and “Seizures”. (C) Relationship between the severity and impact could not be determined for “ Gastroesophageal Reflux”, “Oral Feeding”, “Understanding”, “Verbal Self-Expression”, “Standing Unsupported”, “Walking with Assistance”, “Walking Independently”, and “Seizures”

Core features and impairments in bold

^Impairment in function

## Results

### Characteristics of the RTT subject sample

The majority of caregivers reported on individuals with classic RTT (~ 69%) with a *MECP2* variant (89%). The age range of affected individuals was wide (1.0–61.0 years), with mean and median values of 16.7 and 15.3 years, respectively. Approximately, 40% were children, 20% were adolescents, and 40% were adults. The mean and median age of diagnosis were 4.3. and 2.8 years, respectively (classic RTT mean 3.8 years, median 2.5 years; atypical RTT mean 5.7 years, median 3.9 years), in line with published reports [44, 45]. Approximately 69% of caregivers reported a clinical presentation of classic RTT, ~ 19% reported atypical RTT, and ~ 12% reported ‘don’t know’. Caregiver responses indicated that ~ 55% resided in the U.S.A., ~ 34% in Europe, and ~ 11% in Australia. The country groups only differed in frequency of classic RTT presentation, which was lower for caregivers residing in Australia (~ 54%) as compared to those residing in Europe (75%) or the U.S.A. (68%). For details, see Table 1.

### Frequency of problems

Table 2 depicts the frequency of problems (percentage of caregivers reporting the problem), distribution of categorical responses (i.e., percentage of two highest-level responses in those reporting the problem), and raw and z-score means, medians and SDs for the full dataset. There was a wide range of frequency of problems, from ~ 35% for hyperventilation to 99% for impairments in self-care activities of daily living. Core features or impairments were present in 70–98% of affected individuals, while other problems were more variable. For instance, seizures were reported by 37% of caregivers, dystonia by 53%, and constipation by 79%. Rett episodes (i.e., non-epileptic vacant spells/absences and dystonic crises/episodes) and behavioral abnormalities, which are not included in many surveys of the disorder, were also frequent, both approximately 79%.

### Magnitude of clinical severity and impact

As shown in Table 2 for the primary analyses, frequency and scores on severity and impact were greater for problems representing core features of the disorder and related impairments. Specifically, mean severity and impact z-scores were positive for functional hand use, hand stereotypies, verbal self-expression, standing unsupported, walking with assistance, walking independently, and self-care. Additionally, severity of oral feeding had a positive mean z-score but the corresponding impact scores did not. Of these problems, severity was greater than impact for verbal self-expression, standing unsupported, walking independently, oral feeding, and self-care. For the remaining problems with positive mean severity and impact z-scores, impact on individual was greatest. Positive mean z-scores were also found in impact on caregiver for sleep difficulties, seizures, pain, and behavioral abnormalities.

### Discrepancy between severity and impact

Chi-square analyses demonstrated that, with the exception of understanding, severity and impact responses were statistically inter-dependent (Table 2). Subsequently, Friedman’s ANOVAs showed significant differences between severity and impact scores for all problems but walking with assistance (Table 2). ANOVA post hoc tests showed that there were nine problems that had a significant and disproportionally higher impact than severity while severity and impact scores were comparable for four problems and severity was greater than impact for eight (Table 3). Seven out of nine problems with greater impact than severity affected more caregivers than individuals with RTT; most of them are typically manifested with variable frequency over time. These “episodic” problems include sleep difficulties, seizures, Rett episodes, pain, and behavioral abnormalities. Among particularly impactful problems, only hand stereotypies affected individuals with RTT more than caregivers (Table 3). Figure 1 illustrates different patterns of impact on caregiver.

### Effect of age, clinical type, and country of residence

Secondary analyses showed that score profiles and relationships between severity and impact identified for the entire sample were in general replicated in the age, clinical type, and region group analyses (Tables 4, 5). This was particularly true for problems with greater impact than severity. However, there were exceptions, mainly influenced by subject’s age. Breath-holding, oral feeding, and scoliosis were relatively more impactful than severe in younger individuals, while gastroesophageal reflux, dystonia, and Rett episodes that were less impactful than severe in older individuals. Interestingly, seizures were less impactful for caregivers of adolescents and adults and nonverbal self-expression was more impactful on individuals than caregivers in adults (versus similar impact in children and adolescents) (Table 4). While seizures were particularly impactful in the USA group, their level of impact could not be determined in the European and Australian groups (Table 5). Other significant severity-impact differences were not replicated for some variables in adolescents, individuals with atypical RTT, or in the Australian group, most likely because of the smaller size of these groups.

## Discussion

Rett syndrome (RTT) is a severe neurodevelopmental disorder characterized by a wide and variable range of neurologic impairments and comorbidities, the severity of which can accumulate over time. This study presents an initial overview of BOI in girls and women with RTT. Through an international collaboration of multiple stakeholders, which developed and implemented a caregiver survey targeting 22 RTT-characteristic problems across a large international sample, we were able to identify the most impactful problems, their differential effect on affected individuals and caregivers, and their relationship with clinical severity as estimated by caregivers. We found that among the most frequent, severe and impactful problems were those related to the core features of RTT and related impairments, namely hand function, hand stereotypies, communication and motor impairments, and self-care. We also demonstrated that many problems, particularly those that are episodic in nature (e.g., sleep difficulties, seizures, pain, and behavioral abnormalities), have disproportionately greater impact than severity, affecting caregivers more than individuals with RTT. In the main, these profiles of BOI were not influenced by the affected individuals’ age, clinical type, or country of residence.

Previous studies on quality of life of individuals with RTT and their caregivers have identified multiple factors affecting outcomes. Ability to ambulate, feeding skills, severity of seizures, sleep problems and behavioral abnormalities have impact on quality of life of individuals with RTT [21–23]. Greater severity of child’s impairments, caregiver age and demands, and family function and financial challenges also play a role in caregivers’ physical and mental well-being [25–29, 31]. While these data are extremely valuable, to our knowledge, no study has examined the differential impacts of features characteristic to RTT on affected individuals and their caregivers or the relationship between problem severity and quality of life. Furthermore, the considerable phenotypical variability of RTT [5, 46, 47] makes analysis of large subject samples, as the one employed in this study, imperative in order to obtain representative and reproducible findings.

Problems related to the core diagnostic features of RTT [5], involving communication, fine motor, and gross motor function, as well as self-care, were among the most frequent, severe and impactful manifestations. Our analyses demonstrated an expected interdependence between severity and impact scores, due to the fact that all scoring was conducted by caregivers who were assessing the affected children and themselves. Nonetheless, there was significantly greater impact than severity on both core features (i.e., hand stereotypies, nonverbal self-expression) and common symptoms of mild to moderate severity (e.g., gastroesophageal reflux, sleep difficulties, behavioral abnormalities). This was a novel finding that emphasizes that clinical severity, as estimated by caregivers, may underestimate BOI. Verbal self-expression and self-care seemed to be less impactful than severe, although their overall level of impact and severity were high. Other distinctive RTT manifestations that were particularly impactful on caregivers included seizures, Rett episodes, and pain. As with sleep difficulties and behavioral abnormalities, they were characterized by relatively lower frequency or severity than other problems but also by an episodic nature. Despite this, in evaluations covering only the previous month, caregivers reported they were markedly affected by the occurrence of these problems. Whether their unpredictability contributes to their marked impact is unknown; however, these results agree with informal clinic observations. Our findings also highlight the importance of relatively recently investigated problems in RTT, such as sleep and non-autistic behavioral difficulties [9, 11, 48–51]. They are also in line with studies of individuals with other neurodevelopmental disorders which show that sleep difficulties in children can exacerbate parents’ existing strain and fatigue, adversely affecting their mental health and parenting [30, 52, 53]. The BOI profiles reported here are also in agreement with a recent investigation on top caregiver concerns in RTT, which reported communication, seizures, walking/balance issues, lack of hand use, and constipation as top concerns for caregivers of individuals with classic RTT [54].

Comparisons of impact on individuals and caregivers demonstrated that the latter appear to be more affected by many of the RTT-characteristic problems evaluated in this project. Caregiver’s role in providing daily care, sometimes representing all essential needs of daily living, can be affected by seemingly milder impairments that add emotional, and physical burden and limit time availability for other activities [21, 30, 40]. Our findings are in correspondence to previous studies on morbidity and mortality [19, 20] and quality of life of affected individuals and caregivers [21–23, 25–28, 31, 40] but insights into the wide range of problems and the relative independence of impact from clinical severity for some problems is novel. Indeed, even RTT-characteristic problems that are mild in severity can place a disproportionate burden on affected individuals and, particularly, on their caregivers. Interestingly, the profiles of severity and impact reported for the total subject sample were to large extent replicated in analyses of age, clinical type, and country of residence groups. For instance, the greater impact of seizures on caregivers of younger than older individuals and those residing in the USA may reflect different levels of tolerance for this unpredictable type of symptom.

Despite the large subject sample and multiple countries of origin, our data had limitations. Approximately one third of the submitted entries were determined to be invalid at the initial data quality control phase. This problem is inherent to conducting anonymous online surveys. Among them, assessment of problem severity by caregivers did not follow specific guidelines, there was limited verification of caregivers’ understanding of the survey or of the accuracy of responses about clinical type or genetic variants, and there were structural inconsistencies in the survey (e.g., item severity assessed through 5–7 options). Although analyses included age, clinical type, and country of residence groups, some subgroups were relatively small in size (i.e., adolescents, individuals with atypical RTT, Australian sample), which could prevent the replication of some findings. We acknowledge that we did not have data on specific *MECP2* pathogenic variants, which are known to influence clinical severity but whose effects on impact are not yet known. Additionally, this international survey included caregivers from a range of communities with disparate degrees of exposure to families with children impacted by other serious disorders. Thus, their perception of severity will undoubtably be relative to their specific experience. Therefore, the present report should be considered as an initial overview analysis of BOI in RTT. We expect that follow-up studies will address some of the abovementioned issues by expanding the current analyses. For instance, investigating the role of treatments and their outcomes, healthcare resource utilization, and other factors on RTT burden. Additional collected data on impact on caregiver (health, relationships, financial impact) would further delineate groups particularly impacted by the clinical manifestations of RTT.

## Conclusions

This large-scale study of BOI in RTT demonstrated that the most impactful problems were those related to the core features of the disorder and that even mildly severe clinical manifestations can disproportionately impact affected individuals and their caregivers. Future analyses should explore the influence of other factors such as clinical evolution, treatment outcomes, and access to healthcare services. Similar analyses from the healthcare provider perspective should also expand our understanding of BOI in RTT.

### Supplementary Information

Additional file 1.

## Data Availability

Data are available through Anavex Life Sciences Corp. and the International Rett Syndrome Foundation.

## Abbreviations

RTT: Rett syndrome
MECP2: Methyl-CpG-binding protein 2
BOI: Burden of illness
