# Supplementary material for: Burden of illness in Rett syndrome: initial evaluation of a disorder-specific caregiver survey
Source: Orphanet J Rare Dis. 2024 Aug 13;19:296. doi: 10.1186/s13023-024-03313-8 (PMC11323357; doi:10.1186/s13023-024-03313-8)
Supplement: Supplementary file 1 — Additional file 1. [file 13023_2024_3313_MOESM1_ESM.docx]

**Supplementary Material**

**Examples of Items in the Caregiver Survey**

**Breath-holding**

2. On an average day during the past 4 weeks, how severe was her breath-holding?

- Not at all severe
- A little
- Somewhat
- Quite a bit
- Very severe

3. How much does her breath-holding negatively impact her ability to participate in daily activities? Examples of daily activities include play, hobbies, social events and therapies, both inside and outside of the home (e.g. at school).

- Not at all
- A little
- Somewhat
- Quite a bit
- A lot

4. How much does her breath-holding negatively impact you as a caregiver (e.g. your physical or emotional well-being)?

- Not at all
- A little
- Somewhat
- Quite a bit
- A lot

**Understanding**

1. How would you describe her understanding during the past 4 weeks, taking as a reference a girl of her age without Rett syndrome? *For example, her ability to identify objects or respond to questions (verbally or non-verbally)*.

- Understands everything said to her [Please go to question 74]
- Understands most things said to her [Please go to question 74]
- Understands simple questions and instructions
- Understands some single words
- Understands familiar routines
- No understanding
- Don’t know [Please go to question 74]

1. How much do her limitations in understanding negatively impact her ability to participate in daily activities? *Examples of daily activities include play, hobbies, social events and therapies, both inside and outside of the home (e.g. at school).*

- Not at all
- A little
- Somewhat
- Quite a bit
- A lot

1. How much do her limitations in understanding negatively impact you as a caregiver?

- Not at all
- A little
- Somewhat
- Quite a bit
- A lot

**Epilepsy and Epileptic Seizures**

1. During the past 4 weeks, how many times did she have an epileptic seizure?

- None [Please go to question 107]
- 1-2 times
- 3-6 times
- 7-13 times
- 14-27 times
- 28 times or more
- Don’t know [Please go to question 107]

1. How much do her epileptic seizures negatively impact her ability to participate in daily activities? *Examples of daily activities include play, hobbies, social events and therapies, both inside and outside of the home (e.g. at school).*

- Not at all
- A little
- Somewhat
- Quite a bit
- A lot

1. How much do her epileptic seizures negatively impact you as a caregiver?

- Not at all
- A little
- Somewhat
- Quite a bit
- A lot
